# The UK Longitudinal Linkage Collaboration: a trusted research environment for the longitudinal research community.

**DOI:** 10.23889/ijpds.v7i3.2046

**Published:** 2022-08-25

**Authors:** Andy Boyd, Robin Flaig, Jacqui Oakley, Kirsteen Campbell, Katharine Evans, Stella McLachlan, Richard Thomas, Emma Turner

**Affiliations:** 1 University of Bristol; 2 University of Edinburgh

## Objectives

The COVID-19 Longitudinal Health and Wellbeing National Core Study utilises data from Longitudinal Population Studies (LPS) and whole-population NHS databases: yet no resource existed linking multiple LPS to COVID-19 records. A centralised infrastructure was needed to pool LPS data and systematically link participants’ routine health, administrative and environmental records.

## Approach

The UK Longitudinal Linkage Collaboration (UK LLC) is an unprecedented infrastructure integrating data from major inter-disciplinary and pan-UK LPS and systematically linking these to participants’ COVID-19 relevant records. Integrated and curated data are made available for pooled analysis within a functionally anonymous Trusted Research Environment (TRE). We commissioned a “Secure eResearch Platform - SeRP” to provide an underlying secure computing system including trusted third-party processing of participant identifiers. We developed a bespoke governance and data curation framework designed collaboratively with LPS data managers and with an access mechanism enabling LPS to retain key decision-making controls. Public/participant involvement guided our approach.

## Results

Data from >20 LPS and >250,000 Participants' are linked to NHS records (primary, secondary, community care; COVID-19; civic registers; prescriptions; mental health) and geo-coded environmental exposures (pollution, green space, neighbourhood indicators). UKLLC is now accessible to UK-wide researchers for COVID-19 research. Our pipeline/resource/provision of linked data is based on three key innovations: (1) a “Linkage brokerage” model where our trusted third-party processes participant identifiers for many different data owners; (2) a novel longitudinal data pipeline with NHS records which enables linkage and data extraction and update of records over time; (3) a “Delegated & Distributed Access Framework” enabling UK LLC Data Access Panel to consider applications on behalf of data owners (e.g., the NHS) whilst distributing applications to LPS for approval of appropriate data use.

## Conclusion

UK LLC provides a strategic research-ready platform for longitudinal research: a clear step change from pre-pandemic capability. With sustained investment, and through exploring options to extend linkages and generalise to wider purposes, UK LLC is positioned to inform cross-cutting themes such as understanding health and social inequalities, health-social-environmental interactions, and managing the COVID-19 recovery.

